# Pulmonary Vein Stenosis in Children: A Programmatic Approach Employing Primary and Anatomic Therapy

**DOI:** 10.3390/children8080663

**Published:** 2021-07-30

**Authors:** James A. Kuo, Christopher J. Petit

**Affiliations:** 1 Division of Pediatric Cardiology, Children’s Healthcare of Atlanta, Emory University, Atlanta, GA 30322, USA; kuoj@kidsheart.com; 2 Division of Cardiology, Department of Pediatrics, Columbia University Vagelos College of Physicians and Surgeons, Morgan Stanley Children’s Hospital of New York, BN-263a, Pediatric Cardiology, 3859 Broadway, New York, NY 10032, USA

**Keywords:** pulmonary vein stenosis, congenital heart defect, drug therapy

## Abstract

Pulmonary vein stenosis (PVS) is a difficult condition to treat due to recurrence and progression. In 2017, we developed a comprehensive PVS Program at our center to address the multidisciplinary needs of these patients. We discuss the components of our program and our approach to these patients, using a combination of primary (medical) therapy in addition to anatomic therapy to preserve vessel patency. A multidisciplinary approach to treating these challenging patients is critical.

## 1. Introduction

Pulmonary vein stenosis (PVS) in the pediatric population has been thought of as a rare condition [1]. However, many clinicians are now recognizing the disease to be quite common, particularly among premature infants [2]. PVS has been typically classified as primary PVS and secondary PVS. Secondary PVS is more easily understood, as it follows surgical treatment of total or partial anomalous pulmonary venous connection (TAPVC and PAPVC, respectively). Secondary PVS is seen as a post-operative complication in at least 10% of cases [3,4]. Primary PVS is an idiopathic condition associated with mesenchymal proliferation, leading to pulmonary venous vascular changes, often associated with chronic lung disease [5,6,7]. Both primary and secondary PVS have two unique aspects which conspire to make the disease uniquely malignant: recurrence and progression.

Infants and young children with PVS endure a high exposure to morbidities and mortality, particularly in the first 2 years of life [1,2,5,7]. These patients undergo a number of interventions in efforts to maintain patency of pulmonary veins (PV). Additionally, these patients experience growth failure for unclear reasons.

Recent studies have increased our understanding of the pathophysiology of this disease. Animal models of PVS have demonstrated that anatomic PV narrowing can activate a cascade of cellular and molecular pathways, which then lead to upstream conversion of vascular intima to myofibroblasts, as well as to cellular proliferation [8]. Clinicians have employed several approaches to relieving the obstructions resulting from PVS. Surgical and transcatheter therapies have provided temporary relief, but with disappointing mid- and long-term outcomes.

## 2. PVS Program

In 2017, we developed a comprehensive PVS Program at our center to address the multidisciplinary needs of these patients. Recognizing that patients with PVS require close surveillance and ongoing evaluation of their respiratory status, cardiac function, and growth failure, we developed a team which included input from cardiologists, a nutritionist, and an oncologist, as well as occasionally from our pulmonary hypertension specialists. Based upon our review of prior studies, we felt strongly that immunomodulatory therapy would be a critical component to our PVS Program. We also sought to make uniform the follow-up, surveillance imaging, and reintervention necessary to improve survival for this at-risk cohort [9,10].

## 3. Diagnosis and Surveillance

At our center, most patients with PVS have been identified with echocardiography. However, we have found a concerning false-negative and false-positive rate with echocardiography in neonates and infants with PVS. We believe there are multiple causes for this. False negative rates are likely due to the inability of echocardiography to adequately resolve pulmonary veins and to identify true anatomic obstruction in the setting of elevated velocities in veins where left-to-right shunts co-exist. Furthermore, PVS is a progressive disease. A comprehensive initial echocardiogram evaluating all cardiac structures—including pulmonary veins—may appear to rule out a pulmonary vein anomaly, such as PVS. Subsequent echocardiograms on the same patient may not focus on the pulmonary veins again, particularly if the indication for the subsequent echocardiograms is to evaluate ventricular function, the status of a persistent ductus arteriosus (PDA), or otherwise. It can be difficult to assess in regional PVS as lower Doppler gradients can be seen in affected veins and higher gradients in unaffected veins due to redistribution of pulmonary blood flow [11]. Even the use of more invasive transesophageal echocardiography can be difficult to delineate differences in gradient, especially in the upper veins [12].

**Figure 1:**
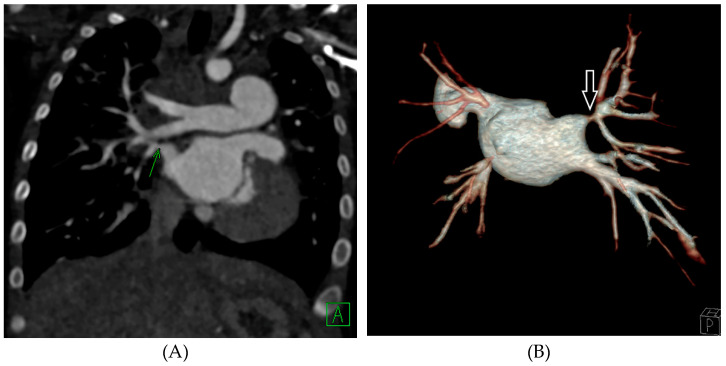
(**A**): Coronal slice of a CT angiogram demonstrates right upper pulmonary vein stenosis (green arrow). (**B**): Posterior view of the three-dimensional reconstruction of the CT angiogram of the same patient also demonstrates right upper pulmonary vein stenosis (white arrow), in addition to severe stenosis of the left lower pulmonary vein and atresia of the left upper pulmonary vein.

Given the limitations of echocardiography, we rely on computed tomographic angiography (CTA) (Figure 1) or catheter-based angiography for definitive diagnosis of PVS. CTA has been preferred over cardiac magnetic resonance imaging (cMR) due to its shorter acquisition time and higher imaging resolution, although potential insights into regional pulmonary blood flow may conceptually be an advantage of cMR [13,14]. Serial CTA for our program has provided insights not only to distal pulmonary artery and vein anatomy, but also airway anatomy and underlying lung parenchyma. Radiation doses from newer CT scanners have been significantly reduced with optimized imaging techniques [15]. We have used serial CTA scans for grading and tracking of progression of all pulmonary veins in each patient. Our PVS Grading scale is listed in Table 1.

Finally, surveillance CTA allows for monitoring of vascular changes, such as upstream progression, in-stent stenosis (ISS), and ostial PV disease.

Cardiac catheterization allows for detailed assessment of both anatomic and hemodynamic status of PVS. In addition to angiography of affected PVs, a full hemodynamic evaluation can reveal the overall status of a patient with PVS. While a typical hemodynamic assessment of vascular stenosis involves measuring the pressure gradient across the stenosis, we have abjured this approach for most patients with established PVS. While gradients may be measured, we focus primarily on right ventricular (RV), pulmonary artery (PA), and left atrial (LA) pressures. The rationale for this is based upon the Gorlin equation: gradient across a stenosis is directly proportional to degree of stenosis, but also proportional to amount of flow [16]. As mentioned above, redistribution of blood flow to unaffected veins can result in gradients which do not reflect actual PVS severity.

**Figure 2:**
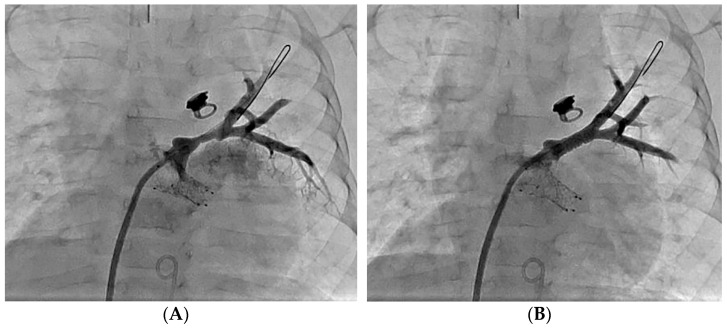
(**A**): Retrograde venography of the left upper pulmonary vein demonstrates proximal stenosis. (**B**): Follow up venography following stent implantation demonstrates improved caliber of the left upper pulmonary vein.

At our center, retrograde venography (Figure 2) in all affected veins is performed to identify stenosis and evaluate upstream arborization. Retrograde venography is performed via a transseptal approach. A large guide catheter (Judkins Right, typically) or Mullins long sheath is advanced to the ostium of the pulmonary vein, and a rapid, low-volume hand injection performed. This retrograde angiogram provides not only detail of the pulmonary vein ostium, but also details of the upstream segmental and subsegmental venous vascular bed.

In contrast, distal pulmonary artery angiography may reveal delayed transit time to the left atrium, but does not provide adequate information regarding pulmonary vein anatomy, particularly when segmental and sub-segmental veins are involved. Further, streaming from unopacified veins during levophase from a pulmonary artery wedge injection complicates the assessment of the pulmonary vein ostium. While this variation in something as simple as angiography may seem trivial, we find that retrograde venography is our gold standard for PV imaging in all cases outside of PV atresia due to the ability to detect disease progression in otherwise unseen feeder vessels.

Catheterization allows for vasoreactivity testing in patients with PVS and pulmonary artery hypertension (PAH). Our center has been more aggressive about using pulmonary vasodilator therapy for patients with elevated PA pressures who have undergone such vasoreactivity assessment [17]. In recent years, patients with RV pressures > 50% systemic at catheterization are given a trial with pulmonary vasodilators while they are in the hospital. If there is no evidence of pulmonary edema or worsening respiratory status, these patients remain on pulmonary vasodilators until RV hypertension and significant PVS are alleviated. At our most recent review, 33% of infants with moderate-to-severe PVS were treated with phosphodiesterase-5 (PDE5) inhibitors.

## 4. Therapy for Pulmonary Vein Stenosis

### 4.1. Primary Therapy

Our typical approach for PVS has evolved significantly over time. This change reflects both the advent of novel therapies and newer devices. Despite the myriad of catheters, stents, and other technical innovations, PVS has been shown to relentlessly return and progress.

More significantly, our approach to PVS has been guided over the past 5 years by the preponderance of translational science which indicates a cellular signaling mechanism, which is responsible for the progression of PVS pathophysiology [8,18,19]. Histopathologic evaluation of PVS samples have revealed intimal hyperplasia and conversion of normal vascular layers to thickened, hypercellular vessels with proliferation of myofibroblasts. Increased expression of transforming growth factor (TGF)-β1 and Smad [18], as well as Ki-67 and phosphor-mammalian target of rapamycin [19], have been described. With new understanding of molecular changes, primary medical therapies have been used to target cellular signaling to reduced anatomic PVS burden. These medications have included vinblastine, methotrexate [20], imatinib, bevacizumab [21], and sirolimus [22,23] with varying results. Losartan has shown some promise in decreasing intimal hyperplasia in a piglet model [24], but its efficacy has not been demonstrated in humans. With these insights, our team has re-envisioned how patients with PVS are managed. We now view disease management with two aims: primary therapy and anatomic therapy.

Primary therapy for PVS entails treating the underlying pathophysiologic process responsible for recurrence and progression of PVS. At our center, primary PVS therapy centers on medications which have promise to alter the cellular signaling pathways believed to be at the core of PVS recurrence and progression. Initially there was some enthusiasm for losartan, an angiotensin receptor blocker (ARB), as animal models of PVS indicated a possible role for ARBs [24]. While there is a very low rate of adverse events with losartan, we found early experience disappointing. Based upon our interpretation of related studies from other investigators, and our own experience with sirolimus for vascular proliferative disease, we chose in 2016 to offer systemic sirolimus therapy (SST) to patients with severe PVS.

When patients are diagnosed with severe PVS, the director of our program (CJP) meets with the family to discuss the risks and benefits to SST. Further, the family meets with our partner in pediatric oncology. If there is parental consent for SST, the patient is initiated on SST following screening laboratory evaluation. The goal level for SST at our center is 8–15 micrograms/dL. Once levels are within this range, patients are monitored monthly. Pneumocystis pneumoniae prophylaxis in the form of Bactrim is given semi-weekly. Further, our team informs the family and the primary pediatrician to avoid live-attenuated vaccines while on SST.

We recently completed a review of the outcomes following SST therapy in 15 patients with severe PVS [25]. We found a positive response in the cohort treated with SST. Interestingly, in this initial study, we reserved SST for those patients with the most advanced, progressive PVS. Even though only severe PVS patients were offered SST, there was a compelling survival advantage among this cohort, suggesting a role for chemotherapy in altering the signal and molecular profile of PVS.

### 4.2. Anatomic Therapy

Transcatheter interventions remain the mainstay of anatomic therapy to preserve PV patency. Our therapies in the catheterization laboratory include conventional balloon angioplasty, drug-eluting stent (DES) implantation, bare-metal stent (BMS) implantation, drug-eluting balloon (DEB) angioplasty, and cutting balloon (CB) angioplasty [26,27,28,29].

Whenever possible, we seek to avoid implantation of coronary stents, as these stents have a maximal diameter of 5.0–5.5 mm. However, when ostial disease is present and is grade 2 or grade 3, a DES is normally implanted to maintain the PV. BMS may be used and are advantageous due to their future expansibility to 8–12 mm in diameter—essentially adult-size. However, they are reserved for PV’s where the upstream vessel is at least 5 mm in diameter. We have found that over-sizing a stent to achieve an arbitrary diameter promotes aggressive ISS and recurrence of PVS. Regardless of the tool employed for an individual PV, close surveillance and repeat intervention appear to be critical to maintaining vessel patency [9]. Our follow-up depends on the severity of the affected PV(s).

For patients with grade 3 (atretic) pulmonary veins, repeat reintervention is normally planned for 1 month from recanalization. This repeat catheterization is performed even in cases in which an echo continues to demonstrate patency. We have found rapid development of upstream progression and ISS in grade 3 veins. For patients whose worst PVs are grade 2 (>50% stenosis), repeat intervention is planned for 6–12 weeks from intervention. Timing of repeat intervention and surveillance catheterization is based upon the interval CTA, the patient’s clinical appearance, and the RV pressure estimated by echocardiography [10]. We have previously reported a treatment algorithm used at our institution [24].

For children whose worst PVs are grade 1 (<50% stenosis), we often employ CTA as interval surveillance, and will determine timing of reintervention based on involvement of other PVs, or appearance of progression within the affected PV.

## 5. Team Approach

Treatment of infants and children with PVS requires the dedication and focus of a multidisciplinary team. In other congenital lesions, the focus on a multidisciplinary team has been found to bring significant benefits to patients [30]. Expertise in cardiac imaging, management of pulmonary hypertension, transcatheter interventions, congenital heart surgery, and oncology-immunomodulation are all necessary for the management of this complex population. Additionally, as most of these patients experience growth failure, it is important that the team identify a champion from nutrition and gastroenterology to work with these patients and their families. Partnership with the primary pediatric team is also critical to ensure that all members of the care team are alerted to changes in clinical status, the need to avoid exposures to ill contacts or live vaccines (in the case of patients receiving SST), and to other concerns. A team-based approach has been advocated to provide consistent care for this complex and recurrent disease [31].

The recurrent and progressive nature of PVS necessitates a sincere attention to the physician–family relationship. A significant investment of time is necessary to appropriately counsel families about PVS and the course of the disease over the lifetime. Continued surveillance can also potentially improve outcomes due to Hawthorne effect [21]. When our team meets with families, we insist on bringing optimism and pragmatism to bear, as so often the family has faced a preponderance of grim, if not outright hopeless attitudes from cardiologists, intensivists, and neonatologists. A belief that PVS is a treatable, if difficult, disease is essential to achieving any success in this field. Once a family understands that PVS can be treated, we begin the conversation about the frequent surveillance and reinterventions that are essential to surviving PVS.

## 6. Conclusions

Our center has found success in treating patients with severe PVS by using a combination of primary (medical) therapy in addition to anatomic therapy to preserve vessel patency. Further study will be needed to demonstrate the ideal combination of therapies to treat patients with PVS. Additionally, we believe that collaboration with experts in genomics and epigenetics will rapidly enhance our understanding of this unique vascular disease process. Finally, while vessel patency and survival are clearly improved with the therapies employed at our and many other centers, these patients continue to require intensive surveillance and frequent interventions, and are often frail and non-thriving. A multidisciplinary approach to treating these challenging patients is critical.

## Figures and Tables

**Table 1:** PVS Grading System.

Grade	A Normal Upstream Vein Caliber	B Hypoplastic Upstream Vein Caliber (<2 mm or <10 mm/m^2^)
**0** Normal Ostium	0	0
**1** <50% Stenosis	1A	1B
**2** >50% Stenosis	2A	2B
**3** Atretic	3A	3B

## Data Availability

No new data were created or analyzed in this study. Data sharing is not applicable to this article.
